# Integration of IoT-Enabled Technologies and Artificial Intelligence (AI) for Smart City Scenario: Recent Advancements and Future Trends

**DOI:** 10.3390/s23115206

**Published:** 2023-05-30

**Authors:** Md Eshrat E. Alahi, Arsanchai Sukkuea, Fahmida Wazed Tina, Anindya Nag, Wattanapong Kurdthongmee, Korakot Suwannarat, Subhas Chandra Mukhopadhyay

**Affiliations:** 1 School of Engineering and Technology, Walailak University, 222 Thaiburi, Thasala, Nakhon Si Thammarat 80160, Thailand; arsanchai.su@wu.ac.th (A.S.); kwattana@wu.ac.th (W.K.); skorakot@mail.wu.ac.th (K.S.); 2 Creative Innovation in Science and Technology Program, Faculty of Science and Technology, Nakhon Si Thammarat Rajabhat University, Nakhon Si Thammarat 80280, Thailand; fahmida_tina@nstru.ac.th; 3 Faculty of Electrical and Computer Engineering, Technische Universität Dresden, 01062 Dresden, Germany; anindya.nag@tu-dresden.de; 4 Centre for Tactile Internet with Human-in-the-Loop (CeTI), Technische Universität Dresden, 01069 Dresden, Germany; 5 School of Engineering, Macquarie University, Sydney, NSW 2109, Australia; subhas.mukhopadhyay@mq.edu.au

**Keywords:** smart city, communication technology, IoT, 5G, AI

## Abstract

As the global population grows, and urbanization becomes more prevalent, cities often struggle to provide convenient, secure, and sustainable lifestyles due to the lack of necessary smart technologies. Fortunately, the Internet of Things (IoT) has emerged as a solution to this challenge by connecting physical objects using electronics, sensors, software, and communication networks. This has transformed smart city infrastructures, introducing various technologies that enhance sustainability, productivity, and comfort for urban dwellers. By leveraging Artificial Intelligence (AI) to analyze the vast amount of IoT data available, new opportunities are emerging to design and manage futuristic smart cities. In this review article, we provide an overview of smart cities, defining their characteristics and exploring the architecture of IoT. A detailed analysis of various wireless communication technologies employed in smart city applications is presented, with extensive research conducted to determine the most appropriate communication technologies for specific use cases. The article also sheds light on different AI algorithms and their suitability for smart city applications. Furthermore, the integration of IoT and AI in smart city scenarios is discussed, emphasizing the potential contributions of 5G networks coupled with AI in advancing modern urban environments. This article contributes to the existing literature by highlighting the tremendous opportunities presented by integrating IoT and AI, paving the way for the development of smart cities that significantly enhance the quality of life for urban dwellers while promoting sustainability and productivity. By exploring the potential of IoT, AI, and their integration, this review article provides valuable insights into the future of smart cities, demonstrating how these technologies can positively impact urban environments and the well-being of their inhabitants.

## 1. Introduction

**Figure 1:**
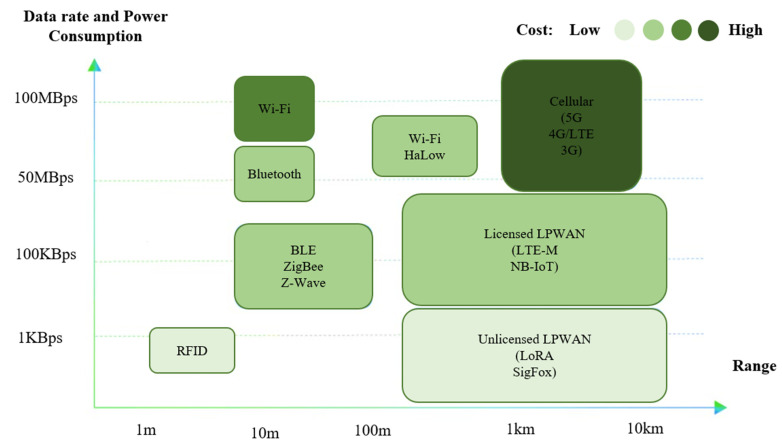
Comparison of date rate, power consumption, cost, and a coverage range of available communication technologies.

Cities are considered complex systems with massive numbers of interconnected citizens, transportation, communication network, varieties of services and businesses, and utilities for improving the lifestyle of urban people. Vast numbers of people are coming towards cities, and the city government is pressured to provide the minimum services required for daily life. The excess population and rapid urbanization bring many problems, such as socio-economic, technical, and organizational problems, and risks to urban cities’ environmental or economic sustainability. Several modern cities faced rapid urbanization by meeting the standards and, in the process, generated pollution, traffic congestion, and socio-economic inequality [1]. Over the past few years, many individuals have migrated towards urban areas, and predictions indicate that by 2030, 60% of the global population is going to be living in urban settings [2]. Due to the increment of people, varieties of smart applications are introduced to make life easier, contributing to smart cities’ development [3,4,5,6]. The smart city concept entails intelligent management of valuable components such as transportation, medical services, utility services, residences, agriculture [7,8], and environmental building [9]. Furthermore, in smart cities, different telecommunication or wireless infrastructures are required to provide the services effectively and connect millions of devices with numerous technologies, such as machine-to-machine (M2M) communication, network virtualization, wireless sensor networks, and gateways [10,11]. Figure 1 illustrates the data rate, power consumption, establishment cost, and coverage range of available communication technologies [12].

IoT plays a dynamic role in the new communication paradigm in our everyday objects, which are equipped with microcontrollers, radio modules, and appropriate communication protocols [13,14]. IoT enables IoT devices to communicate and become essential to smart cities. Many national governments and private organizations use the IoT concept in Information and Communication Technologies (ICT) solutions to manage the idea of a smart city [15]. The IoT concept aims to use community resources better, improving the quality of services (QoS) with reduced operational and management costs in smart cities [16,17]. IoT technologies are essential in developing the landscape of present smart cities and steering the smart city standard to the enormous data scale [18,19]. According to Statista Research in 2022 [20], by 2030, the global count of IoT-enabled devices is projected to exceed 29 billion, nearly three times the figure of 9.7 billion in 2020. The figures mentioned earlier demonstrate that IoT is regarded as one of the most valuable emerging technologies, bringing forth fresh avenues for services, opportunities, and challenges in implementing intelligent applications and offerings. IoT’s significance is intrinsically linked to the advancement and progression of diverse smart city applications, where it serves crucial functions in driving sustainable development and fostering distinctive innovations. Numerous strategies, contexts, technological remedies, and application domains have been put forward to mitigate the complexity and administration of smart cities.

**Figure 2:**
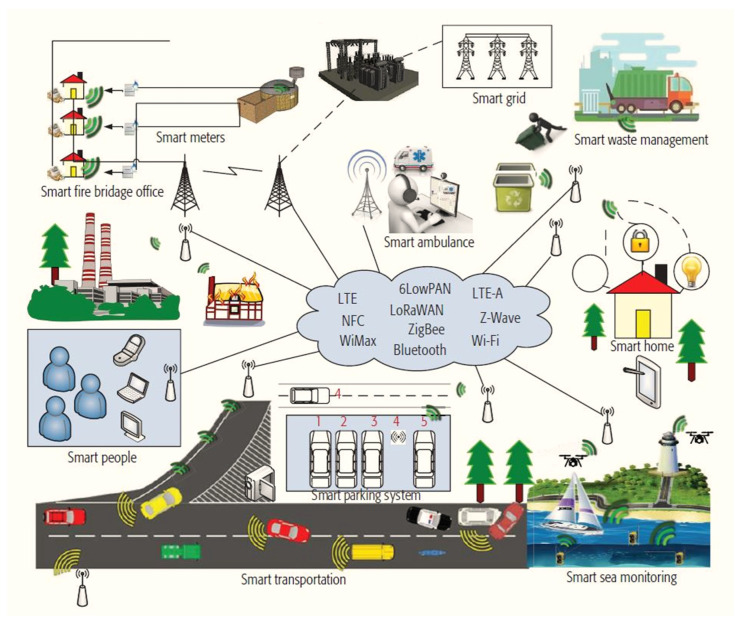
Available communication technologies in various applications for smart cities.

Integrating AI with the IoT in smart cities is a growing trend changing how cities are managed and developed. This integration involves using AI algorithms to analyze the vast data IoT sensors generate in smart cities. This integration also facilitates the advancement of development processes, offering novel opportunities and features, all while significantly reducing human interaction [21]. IoT sensors are devices embedded in different parts of a city’s infrastructure, such as buildings, bridges, roads, and public spaces. These sensors collect and transmit data on various parameters, such as temperature, humidity, traffic flow, energy consumption, and air quality. In general, the use of IoT devices generates vast quantities of data [22,23]. These data subsequently enhance city management and elevate residents’ living standards. However, the sheer volume of data generated by IoT sensors can be overwhelming for human operators to analyze and interpret. AI plays a significant role in this aspect by utilizing machine learning (ML) algorithms to analyze vast amounts of data and detect patterns and trends that might be challenging for humans to discern. Analyzing large amounts of complex data can be challenging to accurately determine the most precise and effective course of action [24,25]. One key area where AI can enhance IoT in smart cities is predictive maintenance. By analyzing data from IoT sensors, AI algorithms can predict when maintenance is needed for city infrastructure, such as bridges and buildings, before a failure occurs. This can help prevent costly and dangerous failures and ensure the city’s infrastructure is always in good condition. AI has enabled various applications such as smart water supply, energy management, waste management, and mitigating pedestrian and traffic congestion, noise, and environmental pollution. Most smart city programs and technologies have focused on collecting large amounts of data and creating solutions that tackle the complexities and dynamics of specific applications [26,27]. AI-powered applications enable the utilization of large volumes of data and knowledge to facilitate decision-making. Around 30% of smart city applications are now significantly integrating AI to enhance urban sustainability, resilience, social welfare, and vitality, including urban transportation solutions. This trend is expected to continue, with an anticipated increase in AI-powered smart city initiatives by 2025 [28]. The rapid expansion of AI-based smart city concepts can be attributed to the relentless drive of researchers, government officials, and urban dwellers to explore new information and methods for building smart cities. The proliferation of automation and AI is becoming increasingly inevitable, with growing demand for their implementation in smart city development [22]. Smart sensor nodes [29] generate large amounts of data associated with various smart city applications and are significantly under-used. Existing Information and Communication Technology (ICT) [30] infrastructure can generate heterogeneous information, which is essential for consolidation. Finally, AI can improve public safety by analyzing data from security cameras, microphones, and other sensors to detect and prevent crime and other security threats. While integrating AI with IoT in smart cities can revolutionize urban development and management, it also raises concerns about privacy, data security, and potential biases in AI algorithms. As such, smart cities must implement strong ethical and regulatory frameworks to ensure these technologies are used responsibly and transparently. The potential threat and concern are not the discussion point of the current review paper. Figure 2 illustrates the communication technologies that are currently available and can be utilized in various applications within a smart city.

IoT-based technologies combined with AI in a smart city can be viewed from various perspectives, including smart sensors, communication technologies, and multiple applications. Among the different technologies, the most critical IoT technologies entail the deployment of reliable and robust networking and communication infrastructures to facilitate efficient data and information exchange among the diverse components of smart city services. Smart city IoT services are designed at varying scales, depending on the type of application. They may require diverse networking and communication technologies for their implementation and operation. Several review papers [16,25,31,32,33,34,35,36,37,38] have previously examined various issues related to big data, network security, and the potential of IoT in smart cities. However, there is a scarcity of resources that comprehensively cover the combination of IoT technologies and the contribution of AI to the smart city concept. This review article discusses the concept of smart cities and the role of IoT in developing them, explaining how IoT enables devices to communicate and become essential components of smart cities. It also highlights how integrating AI and IoT in smart cities can facilitate the advancement of development processes, offering novel opportunities and features while reducing human interaction. The article delves into the potential of AI in analyzing the vast amount of data produced by IoT sensors in smart cities. It highlights how such technology can enhance city administration and ultimately lead to better living standards for residents.

The article is structured as follows: Section 1 is going to provide an introduction to the topic of smart cities, highlighting their significance and potential benefits. Section 2 is going to explain the methodology of searching the relevant article. Section 3 is going to define the concept of smart cities and discuss their essential characteristics and components. Section 4 is going to delve into the IoT-based technologies necessary for creating smart cities, including sensors, networks, and data analytics. Section 5 is going to explore the various AI algorithms suitable for smart cities and discuss their potential impact on urban life. Section 6 is going to examine the possible future trends of smart cities, such as the integration of 5G communication technology and the impact of AI on various applications. Finally, in Section 7, we are going to provide conclusions and prospects for the future of smart cities. Overall, this review article aims to provide a comprehensive overview of the current state and future directions of smart city development, highlighting the potential benefits of this emerging field for urban residents, governments, and businesses alike.

## 2. Methodology

**Figure 3:**
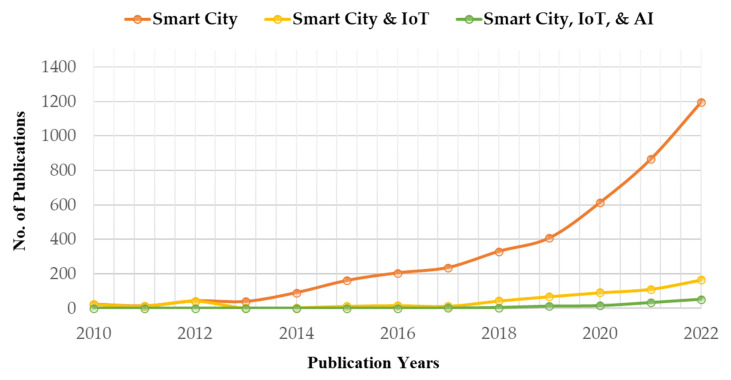
Trends in academic publications on “smart city”, “smart city and IoT”, and “smart city, IoT, and AI” since 2010. The graph presents the number of published articles gathered from a search conducted on the PubMed search engine.

**Figure 4:**
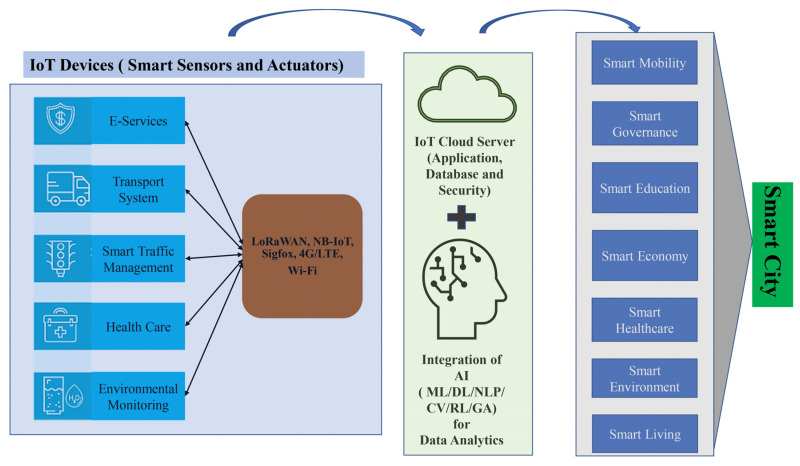
An overview of the methodology for introducing applications in a smart city through integrating IoT and AI.

The field of smart cities is in constant flux, resulting in vast knowledge ideal for an integrative literature review. Relevant academic papers were carefully selected and screened to accomplish this. Two databases, Google Scholar and PubMed, were utilized to gather peer-reviewed articles published by reputable publishers, emphasizing high-quality publications. Additional resources were sourced using the references listed in the collected papers to broaden the scope of the analysis. Academic papers were selected based on their relevance to the investigated issues, ensuring a comprehensive analysis incorporating empirical and qualitative studies. To be included in the analysis, studies had to demonstrate a consistent pattern of findings supporting smart city initiatives’ advancement. Those that presented divergent or inconclusive results were excluded. PubMed was used to search for the keywords “smart city”, “smart city and IoT”, and “smart city, IoT and AI” from 2010, and the number of published articles are gathered and presented in Figure 3. It is evident from Figure 3 that AI, IoT, and smart cities began receiving significant attention after 2015, identifying problems and creating more opportunities in the smart city context. Following a comprehensive analysis of the gathered academic papers, supplementary sources, including reports from governmental agencies, reputable newspaper articles, and websites, were searched on Google to reinforce the emerging conclusions. Figure 4 presents an overview of the methodology for introducing applications in a smart city by integrating IoT and AI.

## 3. The Smart City Paradigm

There exist multiple definitions of the smart city [15,39,40,41]. The term “smart city” is often used interchangeably with other terms such as “intelligent city”, “knowledgeable city”, and “digital city”. This has resulted in diverse theoretical choices for the smart city concept [4]. The term “smart city” is an ambiguous concept used in multiple ways. There is no specific template for defining the smart city, nor is there a unique definition that is universally accepted [42,43]. Smart city concepts emerged as a response to the challenges posed by urbanization and the need for sustainable urban development. Integrating various technologies, such as the IoT, AI, and big data analytics, aims to improve the efficiency of urban services, reduce resource consumption, and elevate the standard of living, which can lead to an improved quality of life for citizens. Technological development has advanced so much that a modern smart city can collect data from various applications, such as smart agriculture, smart industry, smart farming, smart health care, smart traffic, and smart pedestrians, and then analyze and integrate critical data to provide a decision for an improved standard of life [44]. The rapid development of the IoT [45,46], cloud computing technologies [47,48], and AI [49,50] could be vital factors in improving urban facilities’ performance, quality, and interactivity, which is going to decrease management costs and enhance skills.

While the definition of a smart city can vary, there are generally two distinct approaches based on the key areas a smart city should focus on. In the first trend, smart city definitions emphasize a single urban feature, such as technology or ecology, while leaving the rest of the features of a city included. The definitions do not consider the primary aim of a smart city, which is to introduce a novel framework for managing urban areas and assessing the interconnectedness of various components within an urban ecosystem. Focusing solely on the development of a single aspect of an urban system does not necessarily imply that the problems of the entire system can be overcome. It is important to consider the interconnectedness of all urban characteristics to tackle modern cities’ challenges and ensure a thorough and effective approach [51,52,53]. Under a different set of definitions, the focus is on how the smart-city concept represents a comprehensive approach that considers all urban features integral parts of a system. This approach emphasizes that the smart city is a new methodology for organizing and developing a city, which considers economic, technological, and social factors to ensure the stability and sustainability of the urban system as a whole. The descriptions suggest a comprehensive approach to urban issues that leverages new technologies, allowing for a redefinition of the smart city model and the relationships between its stakeholders [54,55,56,57,58]. Table 1 summarizes the definitions of the smart city.

The characteristics of a smart city can be defined as follows:

- The integration of advanced technologies such as IoT, AI, and big data analytics to enhance sustainability, efficiency, and the overall well-being of citizens.
- Implementing renewable energy sources, eco-friendly buildings, intelligent transportation systems, electric vehicles, and efficient traffic management. Moreover, the platform provides insights into various aspects of a smart city, including energy management, smart homes, optimized transportation, smart grid systems, water monitoring, waste management, and streamlined administration. These initiatives aim to monitor existing infrastructure and enhance the quality of life for urban residents.
- Emphasis on citizen engagement and active participation through open data initiatives and digital platforms, fostering transparency and collaboration between citizens and the government.

## 4. The Architecture of IoT-Enabled Smart City

The advancement in technologies could bring millions of intelligent sensors to instrument the infrastructure of the cities, working with speed and network structure equivalent to 1000 IoT devices. Effective management of the data collected from sensors is another crucial aspect. This data’s availability and intelligent management play a vital role in the operation of complex smart city ecosystems, ensuring the accurate functioning of city services. A classification based on the application type is essential to establish a solid foundation for smart cities. As previously mentioned, the degree of smartness in various areas such as governance, economy, healthcare, and others must be evaluated within the context of the smart city application.

**Figure 5:**
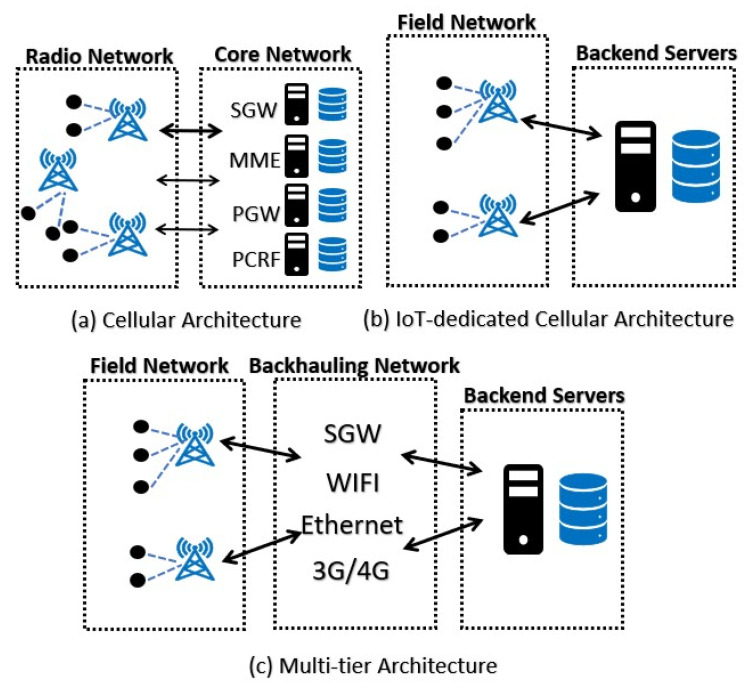
Architectures for supporting the M2M communications in smart cities.

The environment of a smart city is characterized by advanced communication technologies and an IoT infrastructure, which requires specific communication technologies and systems architecture to enable all the applications and advantages of smart cities. The architecture of IoT-enabled smart cities typically requires an ICT infrastructure that facilitates information exchange among the various stakeholders within the urban environment, regardless of the specific application or service being used. Communication is essential to transmit data generated from various sensors in different applications between devices and information sinks in both directions. To accomplish this, three commonly used communication patterns are utilized: (i) utilizing Cellular Mobile Networks for communication, (ii) using IoT-Dedicated Cellular Networks for communication, and (iii) employing Multi-Tier Networks for communication. Figure 5 illustrates the main layout for the three different architectures. Therefore, to achieve a proper architectural ground for smart cities, classification based on the type of application is necessary, as the smartness of entities such as governance, economy, healthcare, etc., must be measured in the smart city application.

The following sections consist of the architectural components with the leading communication technologies available in all three architectural classes in a smart city scenario.

### 4.1. Architectural Components

The literature has discussed five or three components of IoT [32,66,67,68]. However, four major components, such as data sensing/actuating, networking and communication components, vital components, and cloud/fog computing for various services and applications, can be summarized and discussed in the following sections.

### 4.2. Perception or Sensing Layers Components

The components referred to here are the physical objects of electronic devices such as sensors and actuators. These devices interact with the physical world by sending and receiving data, utilizing wireless networks [6,7,8,69,70]. Various smart city applications include sensors, actuators, and smart device technologies in this context. There is a vast array of commercial devices available that can measure different physical quantities and external variables, i.e., sensors for measuring humidity [71,72], temperature [73], environment monitoring [74], water nutrient monitoring [75], etc. Actuators are used for controlling/moving other objects or systems through physical interaction or virtual control [76]. They are usually categorized into pneumatic, electrical, and hydraulic categories [77]. Several software applications and solutions can be used for deploying low-level IoT applications [78].

### 4.3. Networking and Communication

In the context of smart cities, various communication protocols are available to connect different devices and components of the infrastructure. These protocols have additional features and specifications that make them suitable for specific applications. For example, some protocols such as Wi-Fi, ZigBee, and Z-Wave are ideal for short distances where devices and their coverage areas are limited, while other protocols such as LoRaWAN, NB-IoT, Sigfox, and Long-Term Evolution (LTE) are more suitable for long-range applications. Each of these protocols has unique features that enable them to support various smart city applications. For instance, ZigBee is commonly used for low-power applications with a low data rate, offering a secure network and longer battery life. It also provides network topologies suitable for various applications, such as mesh, star, and tree. On the other hand, LoRaWAN, Narrowband-Internet of Things (NB-IoT), Sigfox, and LTE require devices to be connected through a central gateway to collect information and sink the data, and they operate on licensed and unlicensed spectrums, depending on the applications. Therefore, it is essential to choose the appropriate communication protocol based on the specific requirements of each application and the range of coverage needed. Understanding the features and capabilities of different protocols can help smart city planners and developers choose the most suitable protocol for their specific use case and integrate various IoT-enabled devices and artificial intelligence algorithms to create a more efficient, sustainable, and livable city. The subsequent sections are going to discuss some of the significant protocols.

#### 4.3.1. Evolutions of GSM and LTE

The cellular network working groups handle the functions of administration of GSM/GPRS and Edge Radio Access Networks (GERAN). Their efforts are focused on introducing alternative methods to enhance the efficiency of GSM/GPRS for M2M communication. Various methods are available to improve M2M (machine-to-machine) communication efficiency. These approaches comprise upgrading the uplink capacity, extending the coverage for control and data channels, and reducing the power consumption and complexity of M2M devices [79]. Extended Coverage GSM (EC-GSM) is another progressive technique that utilizes Frequency Division Multiple Access (FDMA) in conjunction with Code Division Multiple Access (CDMA) on the uplink to accommodate a more significant number of IoT devices transmitting on the same frequency band. Bind repetition is achieved for all transport channels and extended the coverage regions. It is a technique where the transmitter repeats the same data block several times to achieve higher receiving gains. Different devices are used for different repetition levels in the blind repetition technique. Another approach is to support the narrowband GSM spectrum with channelization of 200 kHz. Narrowband Cellular IoT (NB-IoT) accommodates narrow asymmetric bands in downlink and uplink, where 200 kHz is divided into 48 narrowband channels are downlink channels, and 36 narrowband channels are divided into uplink channels. The Orthogonal Frequency Domain Multiple Access (OFDMA) is employed for the downlink, while the uplink uses Frequency Division Multiple Access (FDMA) to comply with the specific requirements of each device.

One of the evolutions of LTE is LTE Rel-11 [80], which has focused on improving the overload functionalities of RAN for handling the access of many IoT devices with reduced capabilities. The complexity and the cost are reduced by reducing the radio transceiver and limiting the transport block sizes by allowing half-duplex FDD during the operation mode. Moreover, device power saving was also introduced, a significant advantage for IoT applications. Another substantial evolution is the LTE Rel-13 [80] which is improved at the physical layer by including the characteristics of narrowband transmission and provides a better response for M2M requirements. EC-GSM enables the use of cost-effective and low-power hardware while simultaneously enhancing coverage areas. Additionally, Rel-12 has undergone further enhancements and now features an Enhanced Power Saving mode (EPS) and Extended Discontinuous Reception (DRX) functionality.

Cimmino et al. have reported a framework for various applications from the perception of different communication technologies in a smart city scenario [81]. LTE technology has concentrated on leveraging small-cell technology to augment bandwidth within this framework. Small cell technology allows for inexpensive and low-power intelligent devices for various applications. LTE service can be deployed over a larger geographical area and can fulfil all communication necessities, including interoperability, low power consumption, resilient communication, and multi-modal access, which can enhance the overall quality of user experience. Thus, LTE service can be utilized for many applications and may be a superior option for smart cities.

#### 4.3.2. Cellular Mobile Networks

Cellular mobile networks play a significant role in facilitating various services in smart cities. These networks are primarily designed to enable communication between human-to-human and human-to-machine interactions, such as telephony, texting services, multimedia downloading, and streaming [81]. The devices ecosystem also encompasses environment monitoring devices that gather data and require data exchange capabilities with the backend to receive feedback on the monitored data.

The Radio Access Network (RAN) operates on a licensed network, while the Core Network (CN) includes various entities, such as user mobility and registration. For instance, the CN of the LTE system consists of several components, including the Serving Gateway (SGW), the Mobility Management Entity (MME), the Packet Data Network Gateway (PGW), the Home Subscriber Server (HSS), and the Policy and Charging Rules Function (PCRF) server [82]. In smart cities, the Internet of Things (IoT) has ushered in a new communication paradigm known as Machine-to-Machine (M2M) or Machine-Type Communications (MTCs). This paradigm relies on minimal or no human interaction, enabling devices to communicate directly with each other and exchange information without human intervention [83,84].

The characterization of M2M communications are featured distinctively concerning the “legacy” of human-to-human communications, and the end-user human is often replaced by a more significant number of devices, accessing the network periodically or irregularly [85,86,87]. Although 2G/3G (GSM, GPRS) cellular technologies are used for most M2M communications, the RAN and CN of mobile cellular networks face challenges due to the massive M2M data traffic [88]. Hence, extensive research is being conducted to effectively enhance cellular architectures to address the obstacles in M2M communications [87].

As per the guidelines set by the Third Generation Partnership Project (3GPP) [89], the main distinctive features of M2M concerning human-based communication include the following [80]:

- The various market fields, such as smart grid or smart metering, environmental monitoring, and crowd monitoring applications, require different market scenarios to support their diverse applications.
- The end-user device should lower costs with powerful capabilities of handling energy efficiently.
- The number of communication terminals should be large enough to accommodate many devices.
- Every terminal should be able to efficiently handle traffic of varying sizes, ranging from small to large, that originates from the field devices or active smart devices and is transmitted to the network.

In the IoT-based cellular ecosystems, the end-users play an essential role, and the main issues from the user’s side are dealing with cost reduction and network assistive power-saving functionalities, which can improve the lifetime of the devices. From a network perspective, the most critical issues are improving the coverage area and defining lightweight data handling procedures for M2M devices to avoid data overloading difficulties [90]. It is imperative to take action to tackle traffic congestion in both the radio and core network layers [55] and manage the interplay between M2M communication by effectively allocating radio resources. The GSM [91] and LTE standards [92] are some of the initiatives launched by 3GPP to address these challenges [93]. An outline of the primary characteristics of upcoming standardization endeavors in cellular IoT is provided in Table 2.

#### 4.3.3. IoT-Dedicated Cellular Networks

This cellular network tries to design low-cost, low-energy, and low-traffic congestion IoT devices with reduced traffic requirements. IoT-dedicated cellular networks provide many advantages, including reduced power consumption and Total Cost of Ownership (TCO) compared to traditional cellular networks, as well as worldwide coverage and hassle-free plug-and-play connectivity. The network architecture follows a common star topology with extensive coverage areas and can accommodate numerous dedicated devices for applications such as smart lighting, smoke alarm systems, environmental monitoring, smart parking systems, and smart pedestrian counting. These applications may not be feasible with traditional GPRS/GSM networks due to their higher costs, subscription fees, and power consumption [96]. The key distinguishing factors among IoT-specific cellular networks are their bandwidth, ability to handle bi-directional traffic, and network availability. Table 3 provides a comparison of the primary technologies and their respective features.

##### Sigfox Protocol

Sigfox [97] adopts an ultra-narrowband (UNB) technology where devices communicate directly to the base station in a star-like topology. Multiple base stations are available for data collection, making it a reliable network. Most devices are used for uplink communication, and tiny downlink channels control the device from gateways. Sigfox network supports a message payload capacity of up to 12 bytes and employs a technique where the same payload message can be transmitted multiple times for robust reception. The frequency of messages a device can transmit daily can be customized based on the application’s specific requirements. The network coverage of Sigfox is estimated to reach up to 10 km in urban areas and 50 km in rural areas. Building, installing, and maintaining the low-powered network lies with Sigfox Network Operators (SNO). They are also responsible for business development and maintaining/upgrading the reference architecture if necessary [97].

##### Weightless Protocol

**Figure 6:**
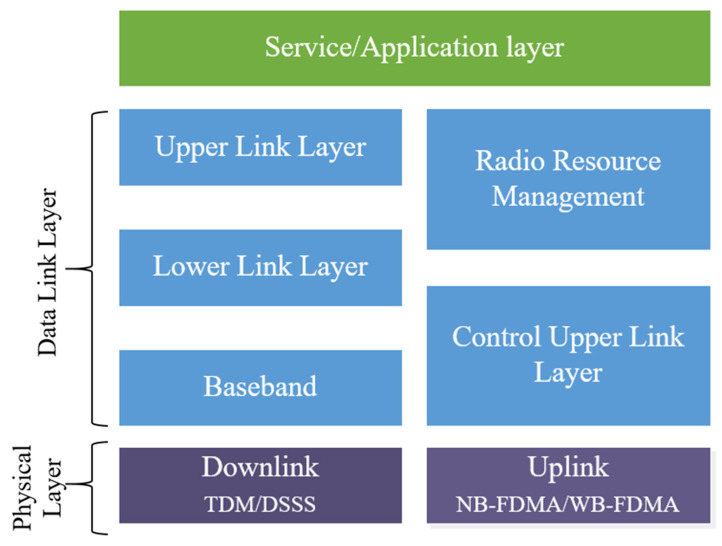
The reference architecture for the weightless system.

The weightless system is controlled by the Weightless Special Interest Group (SIG) [98], a nonprofit organization for implementing the standard architecture. The reference architecture of the weightless system is illustrated in Figure 6. It includes the uplink and downlink, which are part of the physical layer. The uplink transmission adopts FDMA to enable concurrent transmissions and prevent interference. The base station also uses a time scheduling method to notify the devices of their time allocation.

The data link layer comprises three sub-layers: the baseband, lower link layer, and upper link layer. The management of radio resources at the Medium Access Control (MAC) layer and configuration of network arrangements are handled by the Radio Resource Manager (RRM). Additionally, the system supports transmission acknowledgement, fragmentation, and multi-cast and can interrupt the base station.

##### Low Power, Wide Area Networking (LoRaWAN)

The LoRa Alliance [1] has developed a standard for connecting low-power IoT devices covering extensive areas. The standard uses different ISM bands according to the regulations in specific regions. Various ISM bands comply with specific regional regulations in the standard. The physical layer incorporates Chirp Spread Spectrum (CSS) modulation, facilitating two-way communication between base stations/gateways and end devices [41]. Three modes are available in the Medium Access Control, enabling access control while balancing the uplink and downlink capabilities.

**Figure 7:**
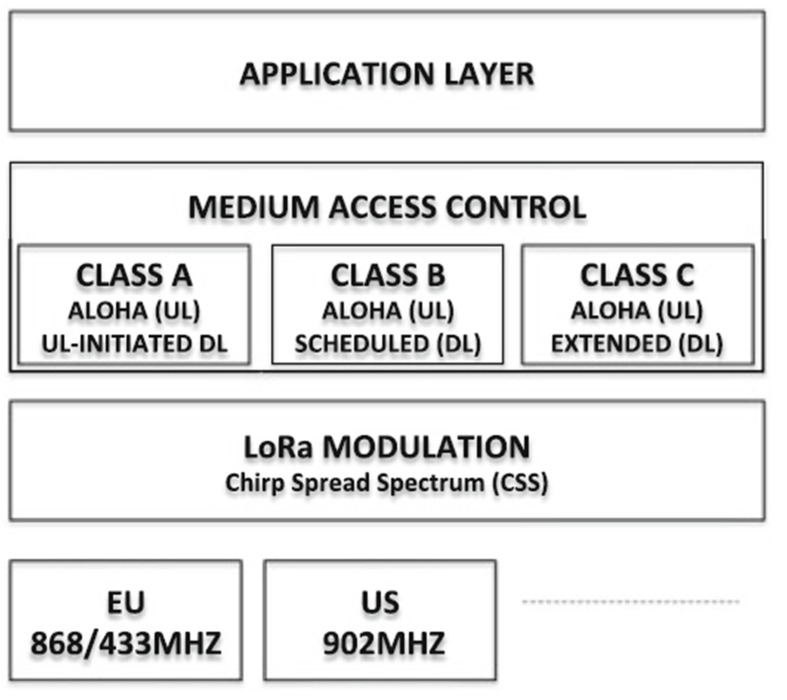
The reference architecture of LoRaWAN.

Figure 7 illustrates the reference architecture of the LoRaWAN. The Class A device requires low power and limited downlink channels for communication from the base station, and Class B shares the same characteristics. However, they have extra time windows not available on Class A devices. Class C device has continuous receiving time windows. It can support multicasts, such as Class A and Class B. During communication between the base station and the end devices, all classes of devices in LoRaWAN utilize the ALOHA access protocol.

### 4.4. Communications in Multi-Tier Architectures

Multi-tier architectures [99] rely on a layered approach to collect and process sensing data and establish a network infrastructure in a multi-hop manner. The sensor data are typically transmitted to central gateways, which then transmit the data to the internet using various communication technologies. The multi-hop transmission technique [100,101] compensates for the limited communication ranges by introducing various radio technologies. This technique also helps to use an extremely low-powered device, which is essential for specific applications. On the other hand, a number of devices can be utilized due to the limited radio technologies needed to ensure connectivity. Table 4 compares several short-range communication standards for their use in multi-tier architectures [102]. Such architectures are based on a layered design and utilize multi-hop routing to establish a network infrastructure.

The IEEE 802.15.4 standard [103] is designed for low-cost, low-data rate, and low-power consumption personal wireless networks. The protocol stack comprises PHY and MAC layers, and it supports operation on three unlicensed frequency bands, namely 868 MHz, 915 MHz, and 2.4 GHz. The standard uses the direct sequence spread spectrum (DSSS) modulation scheme, enabling 20, 40, and 250 kbps data rates on the three frequency bands. Over time, the IEEE 802.15.4 standard [103] has undergone various enhancements to improve its performance, especially in the PHY layers. The MAC layers employ carrier-sense multiple access with collision avoidance (CSMA-CA) mechanisms to facilitate channel access, synchronization, PAN association, and beacon transmission of devices.

Numerous solutions have emerged from the IEEE 802.15.4 standard [103], such as ZigBee, 6LoWPAN, WI-SUN, and ULP, which can be used for diverse applications in personal wireless networks. The Z-Wave Alliance developed the Z-Wave protocol stack for home automation applications. It utilizes Gaussian frequency-shift keying (GFSK) modulation and mesh network architecture for routing and security. The Z-Wave protocol stack defines all the available layers, and one company operates it to enable interoperability between companies. Variable data rates of 9.6/40/100 kbps are possible, and its communication range is similar to IEEE 802.15.4-based solutions.

The Metering Bus (M-Bus) is a specialized bus-based transmission technique for metering utility services such as gas, electricity, and water. It is essential for developing smart utility services and can operate in three frequency bands (169/433/868 MHz) with star or mesh network topologies. It employs synchronized time-division multiple access (TDMA) routing protocols and can communicate up to a range of 300 m with high data rates of 100 kbps.

#### Bluetooth Low Energy and Wi-Fi Low Power

Bluetooth Low Energy (BLE) has recently been very popular in audio streaming and is suitable for IoT applications. It is a low-powered protocol stack where the BLE devices consume low energy and can be used for a long time. The first modulation scheme is GFSK, with a 1 Mbps data rate. There are 40 different channels; 3 are used for advertising, and 37 are data channels. The BLE can integrate with IPv6 and supports the packet fragmentation technique with basic security features. BLE’s major disadvantage is that it only supports mesh topologies, which limits the applications to real-life scenarios. Wi-Fi Low Power increases Wi-Fi range by using less power and operating in the 900 MHz frequency spectrum. As a result, it is appropriate for IoT applications. The data rate is increased from 150 kbps to 8 Mbps by using the OFDM waveform scheme in conjunction with Binary Phase Shift Keying (BPSK), Quadrature Phase-Shift Keying (QPSK), and Quadrature Amplitude Modulation (QAM) modulation. It is also possible to add more devices by using the MAC layer. It is a better protocol for various applications because it has a power conservation mode, a data optimization method, and an extended sleeping state.

Khorov et al. [104] have reported a work that is based on Wi-Fi technology (IEEE 802.11 [105]). They found that Wi-Fi technology [106] has been extensively exploited in applications for smart cities. Due to the abundance of gadgets connecting, there is a severe interference issue. An anti-interference mechanism is essential to reduce the interference effect, and Wi-Fi can be more useful in IoT-based smart cities [107]. Duarte et al. [108] have reported a mechanism to measure Wi-Fi signal strength and developed smart medical health care for smart city applications. Every patient in a medical hospital carries a smartphone with Wi-Fi connectivity. The number of patients waiting in the waiting area, where they are located, and their real-time movements are all determined by the signal strength data gathered from their cell phones. The system’s main benefit is that it can be implemented in the current infrastructure without any new infrastructure. The information gathered can be used to spot future medical problems, which is helpful for doctors.

## 5. AI on Smart City Technologies

Artificial Intelligence (AI) can revolutionize the technology used in smart cities by facilitating instant analysis of vast amounts of data received from various sources, including sensors, cameras, and IoT devices. AI optimizes and streams various city systems and processes, including transportation, energy, public safety, health care, education, and more [109]. No specific number of AI algorithms can be used in a smart city. The choice of algorithms would depend on the smart city’s specific use cases and requirements. Several commonly used artificial intelligence (AI) algorithms exist in smart cities [110]. Some of the most important ones are explained in the subsequent sections.

### 5.1. AI Algorithms in Smart Cities

Various AI algorithms can be used in smart cities to improve efficiency, sustainability, and quality of life. Some of the primarily used AI algorithms are discussed in the subsequent sections.

#### 5.1.1. Machine Learning (ML)

The utilization of ML algorithm [111,112] involves mathematical guidelines that facilitate machines to learn from data and enhance their performance in a particular task without being explicitly programmed. This type of AI empowers machines to identify patterns and make predictions using input data. The ML algorithms comprise supervised learning, unsupervised learning, reinforcement learning, and deep learning, each with advantages and disadvantages, and can be applied to diverse domains, including image and speech recognition, as well as predictive analytics [113,114]. ML algorithms can be utilized in smart cities to examine data and create projections based on patterns and trends humans can discern [115]. These algorithms can optimize resource allocation, improve urban planning, and enhance public safety, among other things. These algorithms can also predict traffic flow, analyze energy consumption patterns, and predict crime hotspots. ML can be used for various applications in smart cities, such as traffic management, energy optimization, and waste management.

#### 5.1.2. Deep Learning (DL)

DL refers to a category of ML that employs artificial neural networks to address intricate problems by acquiring knowledge from data. DL models have multiple layers of interconnected nodes that process specific aspects of the input data and pass it on to the next layer [116,117]. This enables them to acquire a hierarchical understanding of the data, detecting intricate patterns and characteristics. Widely used DL structures comprise convolutional neural networks (CNNs), deep belief networks (DBNs), and recurrent neural networks (RNNs). DL can be applied in smart city scenarios to optimize traffic flow by predicting traffic patterns and tracking vehicles and pedestrians using real-time analysis of traffic camera feeds. It can also predict energy demand, identify energy-efficient building settings, optimize waste collection, and reduce landfill waste using data from sensors and cameras [118,119]. DL can also detect and respond to safety threats using data from surveillance cameras and improve the ability of autonomous vehicles to detect and respond to objects and obstacles [120]. While DL models demand significant data and computational resources, their implementation can enhance the efficiency, sustainability, and safety of smart cities for the inhabitants.

#### 5.1.3. Natural Language Processing (NLP)

The subfield of AI, known as NLP, focuses on using natural language to facilitate communication and interaction between humans and computers [121,122]. It is a field of computer science that aims to enable computers to comprehend and produce human language, both in spoken and written forms. NLP algorithms are specifically developed to process and analyze vast amounts of natural language data, including texts, speech, and even emojis, with the ability to carry out various tasks such as sentiment analysis, language translation, speech recognition, and text summarization [122]. NLP has multiple applications in smart cities. For instance, it can be used to power chatbots and virtual assistants to handle customer service, analyze social media data and other text-based sources to identify potential safety threats, analyze public opinion on various urban planning initiatives, provide multilingual support, and analyze social media data to identify traffic issues and provide real-time updates to drivers [123,124]. NLP has the potential to make smart cities more efficient, effective, and responsive to the needs of residents by reducing the workload of human customer service representatives, improving the overall customer experience, improving traffic flow, and helping city planners make more informed decisions [125,126].

#### 5.1.4. Computer Vision (CV)

In AI, CV algorithms encompass a range of mathematical and computational techniques that empower machines to analyze and comprehend visual data obtained from their surroundings [127,128,129]. These algorithms can recognize patterns and features in images, videos, and other visual data and use that information to make decisions and predictions. CV is a crucial area of research in AI and is used in a wide range of applications, including facial recognition, object detection, image segmentation, and autonomous vehicles. Some of the most commonly used CV algorithms include convolutional neural networks (CNNs), widely used for image and video recognition, and object detection algorithms, which can detect and identify objects in images and videos. Other CV algorithms include feature detection algorithms, which can identify specific visual features in an image, and segmentation algorithms, which can separate an image into different regions or objects. CV algorithms have many smart city applications that can help improve traffic management by analyzing camera feeds and giving real-time updates to drivers, detecting public safety threats through analyzing surveillance camera feeds, optimizing waste collection routes and reducing resources spent, monitoring energy consumption in buildings and identifying areas to promote energy efficiency and understand development patterns and land use in urban planning initiatives through satellite imagery analysis. By automating visual data analysis from various sources, CV algorithms can make smart cities more efficient, safe, and sustainable [130,131,132].

#### 5.1.5. Reinforcement Learning (RL)

RL is a type of ML that involves an agent learning through trial-and-error interactions with its environment to maximize a cumulative reward signal [133]. The agent takes action in the background, and the environment responds with a positive or negative reward signal, and the agent learns from this feedback. The RL algorithm involves three components: the environment, the agent, and the reward function. RL techniques include value-based, policy-based, and actor-critic methods. RL has been applied in various fields, including game playing, robotics, and autonomous systems. In smart cities, RL can optimize traffic and energy management systems [134,135,136,137]. However, RL can be computationally intensive and requires much data to train.

#### 5.1.6. Genetic Algorithms (GA)

GAs are optimization algorithms that draw inspiration from the principles of natural selection. These algorithms are frequently employed in AI and ML to tackle complex optimization challenges that traditional methods cannot address [138]. In smart city applications, GAs can optimize many systems and processes, such as traffic, energy, waste, and urban planning. For example, GAs can optimize traffic flow by finding the best timing for traffic lights or the most efficient routes for public transportation. They can also be used to optimize energy consumption in buildings by finding the most efficient settings for heating, ventilation, and air conditioning systems. One potential challenge of using GAs in smart city applications is the computational complexity of the optimization problem [139,140,141]. The problem’s size and the fitness function’s complexity can make finding an optimal solution in a reasonable amount of time challenging. Therefore, it is essential to carefully design the problem formulation and choose appropriate GA parameters to balance the exploration and exploitation of the solution space.

These are just a few examples, and many more AI algorithms can be used in a smart city depending on the specific needs and goals of the city.

### 5.2. Potential Influence of AI on Smart Cities

The impact of AI on smart city technologies is transformative. Implementing artificial intelligence (AI) in urban areas can significantly improve people’s quality of life and revolutionize critical aspects of a smart city. According to various sources, including [19,142], AI can positively impact seven major domains: smart mobility, smart governance, smart education, smart economy, smart healthcare, smart environment, and smart living. The following sections are going to elaborate on the potential influence of AI in each of these domains.

#### 5.2.1. Smart Mobility

Smart mobility involves using advanced technologies and innovative transportation solutions to improve transportation systems, making them more efficient, sustainable, and accessible. This includes integrating digital technologies such as AI, IoT, data analytics, and automation to optimize transportation networks and enhance the mobility of people and goods. By incorporating these technologies, transportation systems can become more efficient, safer, and sustainable. AI and smart mobility have a symbiotic relationship where AI enables smart mobility solutions to operate more effectively and efficiently, particularly in areas such as traffic management, autonomous vehicles, and predictive maintenance. Integrating AI and smart mobility is crucial for improving transportation systems and the mobility of people and goods.

H. Yang et al. [143] proposed a DL model to predict high-resolution traffic speed by combining traffic flow dynamics with topological interdependence. S. Wang et al. [144] introduced a method for traffic signal control that utilizes deep RL and high-resolution event-based data. M. Hassan et al. [145] suggested a congestion-free traffic management approach for smart cities that employs a multi-layer Extreme Learning Machine (ELM) within a DL framework. P. Kaur et al. [146] conducted a methodical review of AI techniques for multi-plate multi-vehicle tracking systems. The review assessed various methods and algorithms employed for vehicle detection, tracking, and recognition, encompassing deep learning-based approaches.

G. Bathla et al. [147] presented an overview of the applications, challenges, and opportunities of autonomous vehicles and intelligent automation. The study argued that autonomous vehicles have the potential to revolutionize transportation by enhancing safety, reducing traffic congestion, and improving mobility for all. Y. Ma et al. [148] provided an overview of validation and verification techniques used for the decision-making and planning of autonomous vehicles. G. Bendiab et al. [149] deliberated on the potential security and privacy risks of the advent of autonomous vehicles (AVs) and emphasized the significance of addressing these concerns to realize the full benefits of Avs. L. Hernandez and A. Castillo [150] examined cloud computing applications in intelligent vehicles and highlighted the advantages of cloud computing, including enhanced storage capacity and computational power, real-time data analysis, and remote access to vehicle data.

For predictive maintenance, C. Yun et al. [151] present an extensive analysis of predictive maintenance in the context of smart city planning. They define predictive analytics as using statistical techniques, ML algorithms, and data mining methods to examine historical and real-time data and make projections for future events and trends. S. Kussl and A. Wald [152] review smart mobility and its implications for road infrastructure provision, emphasizing predictive maintenance and its potential effects on road infrastructure provision.

#### 5.2.2. Smart Governance

Smart governance is a fundamental aspect of smart cities, which aims to transform traditional governance models by leveraging advanced technologies to provide better services and decision-making processes. With the help of digital technologies such as AI, blockchain, IoT, and data analytics, governments can optimize their operations, streamline decision-making, and provide better services to their citizens. Examples include AI-powered chatbots and virtual assistants, which can automate mundane tasks and respond promptly and precisely to citizen queries. Blockchain can enhance government operations’ security, transparency, and traceability, including voting systems, identity management, and supply chain management. IoT sensors within smart cities allow for acquiring real-time information on essential urban factors, including traffic patterns, air quality, and waste management. This abundance of data has the potential to assist governments in making informed decisions and enhancing their services to better cater to the requirements of their constituents. In general, intelligent governance is critical in fostering citizen participation, boosting transparency and accountability, and elevating the quality of life within urban areas.

S. A. A. Bokhari and S. Myeong [153] explored the connections between AI, social innovation (SI), and smart decision-making (SDM) in South Korea and Pakistan. They learned about a favorable and robust mediating effect of SI between the association of AI and SDM, indicating that SI plays a crucial role in AI decision-making. G. V. Pereira et al. [154] reviewed and proposed that smart governance can be regarded as the intelligent utilization of ICT for enhancing decision-making and cooperation between various stakeholders, such as citizens and government. ICT tools, such as social media, can potentially augment citizen engagement and facilitate the establishment of novel governance models for smart government. A. Kankanhalli et al. [155] investigated the potential benefits of employing AI and the Internet of Things (IoT) in smart governance to improve decision-making and provide valuable services in various domains, including transportation, energy, healthcare, education, and public safety.

In a study by M. K. Hasan and colleagues (2019) [156], a blockchain-based Internet of Things (IoT) framework was proposed to address issues of price hikes and corruption in Industry 4.0 and blockchain 5.0. The framework utilized a remote database integrated with blockchain technology, allowing government authorities to monitor transactions between industrial companies and purchasers. Another study by C. van Noordt and G. Misuraca (2020) [157] found that while AI is commonly used to enhance public service delivery and internal management, its use in policy decision-making is still limited.

L. Anthopoulos et al. [158] investigated the relationship between Smart Governance (SG) and Smart City (SC) and examined their significance to scholars in public administration, political science, and information science, as both topics are becoming increasingly popular. Ž. Bojović et al. [159] presented a fresh approach to modelling information systems to assist public administration in the transition from e-government to smart government. To overcome this challenge, the authors proposed using an integration layer for existing databases and services and recommended the application of innovative technologies to facilitate better problem-solving, optimal resource utilization, and policy innovation. Meanwhile, Al-Besher and Kumar A (2020) [160] discussed the challenges governments face in providing e-government services to citizens. They argued that the efficiency and effectiveness of such services could be improved by leveraging AI and IoT technologies. Similarly, Ahn and Chen (2019) [161] suggested that training government employees on AI technologies could enhance their understanding and appreciation of the technology, promoting a culture of innovation and supporting meaningful and sustainable digital transformation.

#### 5.2.3. Smart Education

Smart education involves the integration of advanced technologies and novel teaching approaches to boost the effectiveness of learning and elevate the standard of education. Its primary objective is to leverage technology to enhance educational quality, promote better student performance, and create an interactive and engaging learning experience that is both efficient and effective. In a smart education system, students have access to customized learning content tailored to their individual needs and abilities, and teachers have access to data-driven insights that help them understand how their students are learning and where they may need additional support. Smart education also involves the integration of various technologies and devices, such as interactive whiteboards, tablets, and educational apps, to create a more dynamic and collaborative learning environment.

According to J. Qu et al. [162], incorporating personalized learning as a significant element can drive educational innovation and development. They propose that integrating AI into education can lead to a new education model, resulting in better learning outcomes. AI education is being developed at different rates in different countries, but there is an increasing demand for AI training and the establishment of platforms to support it. A. Bhutoria [163] conducted a literature review on the application of AI in personalized education and identified key themes that suggest structural modifications to the current education system. In a systematic review of literature conducted between 2019 and 2021, covering China, India, and the USA, over 2000 search results were obtained and filtered down to 353 relevant papers using an NLP model. The review revealed that AI has effectively addressed students’ learning needs, habits, and abilities by guiding them through optimized learning paths. In addition, K. Zeeshan et al. [164] have presented a comprehensive overview of the potential applications of IoT in education for school management, teachers, and learners. The authors reviewed recent research on IoT applications in education and discussed how the technology could benefit these three groups.

A. Alam [165] investigated how AI can be applied in education, including adaptive learning, smart campus management, intelligent tutoring robots, teacher evaluation, and virtual classrooms. After assessing the influence of AI technology on education, it has been determined that it has a beneficial impact on both the caliber of teaching delivered by educators and learners’ academic achievements. Y.-H. Hu et al. [166] investigated the application of Robotic Process Automation (RPA) and predictive analytics in developing an Intelligent Tutoring Robot (ITR) aimed at students engaged in distance learning. The ITR is designed to provide automated responses to students utilizing NLP, knowledge representation, ML, inference, Rapid Domain Adaptation, and large-scale parallel computing. It also continuously learns and enhances its capabilities. A. Ni and A. Cheung [167] analyzed the implementation of intelligent tutoring systems (ITS) for English language learning in secondary schools in China. The study concluded that the model has significant explanatory power and can be utilized in future research on ITSs in K-12 education.

In their work, W. Yang [168] examined the obstacles and factors that must be considered while creating an AI syllabus for young children in early childhood education. According to the proposed model, the fundamental AI concepts that can be introduced to young children include how AI algorithms can be persistently trained to detect patterns, make predictions, and provide recommendations, despite constraints. J. Su and Y. Zhong [169] proposed three proficiencies crucial for AI literacy: AI Skill, AI Knowledge, and AI Attitude. They suggested that employing a social robot as a learning companion and programmable tool can effectively teach young children the principles of AI. L. Chen et al. [170] conducted a comprehensive evaluation and analysis of the impact of AI on education, with a particular focus on its implementation and effects on administration, instruction, and learning. The findings of the study suggested that AI has the potential to enhance the quality of education and improve student outcomes significantly.

#### 5.2.4. Smart Economy

In a smart city, the concept of a smart economy involves the integration of advanced technologies and data-driven solutions to create a sustainable and innovative economy. This can include the use of AI and big data analytics to optimize business processes, as well as the implementation of smart payment systems and blockchain technology to enhance financial transactions. Moreover, a smart economy in a smart city can also involve the development of new industries and business models centered around innovation and sustainability, such as green energy, circular economy, and the sharing economy. By leveraging advanced technologies and data-driven solutions, businesses can create new products and services that are more efficient, sustainable, and customer-centric, thereby driving economic growth and competitiveness. In addition, a smart economy can also help promote entrepreneurship and job creation by providing access to training and funding programs and supporting the development of innovation ecosystems that foster collaboration and knowledge sharing among businesses and entrepreneurs. Overall, an innovative economy is a critical component of a smart city, as it enables sustainable economic growth, promotes innovation and entrepreneurship, and enhances citizens’ overall quality of life.

The notion and functioning of smart factories are introduced and contrasted with conventional factories by R. Benotsmane et al. [171]. The article emphasized smart factories’ economic and social operational prerequisites and effects. A. Aliahmadi et al. [172] proposed a theoretical structure for a sustainable supply chain based on Artificial Intelligence of Things (AIoT) and examined its crucial aspects, constituents, and indicators. The framework applies to various industries and practices, and comprehending its dimensions can assist in its implementation. M. Amiri-Zarandi et al. [173] suggested using a platform approach, which considers six fundamental necessities for smooth integration, handling, and utilization of agricultural data: interoperability, scalability, reliability, end-to-end security and privacy, real-time data processing, and standardized regulations and policies. Implementing a smart farming platform that fulfils these requirements can enhance connected smart farms’ productivity, profitability, and overall performance. Y. Zhou et al. [174] examined the utilization of IoT and AI technologies in greenhouse agriculture to enhance productivity and resource management. This approach has proven to be beneficial in meeting the requirements of greenhouse agriculture and fostering innovative agricultural product development practices.

D. Valle-Cruz et al. [175] investigate the potential of implementing AI in public budget allocation to enhance economic, political, and social outcomes. The researchers propose an algorithmic approach utilizing the multilayer perceptron and a multi-objective genetic algorithm to examine World Bank Open Data from 1960 to 2019 across 217 countries. The findings indicate that AI techniques can aid in distributing public spending to increase GDP, decrease inflation, and decrease the Gini index. G. Tran Thi Hoang et al. [176] analyze 76 articles to create a comprehensive map for applying various decision-making methods in different decision and implementation phases. The article recommends that AI could enhance multi-stakeholder involvement in decision-making procedures.

In their study, I. M. D. Andrade and C. Tumelero [177] used data content analysis with Atlas.ti software to analyze how AI can improve customer service efficiency and provide insights on the potential benefits of AI in enhancing customer experience and driving innovation in service processes and the economy. Meanwhile, M. Qin et al. [178] offer valuable insights for AI developers and e-commerce platforms aiming to improve online customer service strategies. Finally, to enhance their resilience and adapt to changing circumstances, companies should consider AI and other emerging technologies, as suggested by P. Agarwal et al. [179], who examined the effects of COVID-19 on these industries and discussed how information technology could be used to implement business strategies to transform businesses and incentivize the implementation of these technologies during emergencies and suggested that companies should consider AI and other emerging technologies to enhance their resilience and adapt to changing circumstances in the future.

#### 5.2.5. Smart Healthcare

Smart healthcare in a smart city encompasses a range of innovative technologies and solutions that aim to improve access to healthcare services, enhance patient outcomes, and increase the efficiency of healthcare delivery. These technologies include the Internet of Things (IoT), Artificial Intelligence (AI), machine learning, wearables, and telemedicine. Smart healthcare enables patients to receive personalized and real-time care, regardless of location. Patients can access healthcare services remotely through telemedicine and wearable devices, enabling healthcare professionals to monitor their health conditions continuously. Smart healthcare also facilitates collecting and analyzing large amounts of patient data, providing valuable insights into patient health trends and treatment efficacy. Moreover, smart healthcare in a smart city also includes using AI-powered chatbots and virtual assistants that can triage patients, provide medical advice, and schedule appointments, reducing the burden on healthcare professionals and improving the efficiency of healthcare services. Smart healthcare solutions offer immense potential to revolutionize healthcare delivery in a smart city, from improving patient outcomes to reducing healthcare costs and increasing access to healthcare services.

P. Manickam et al. [180] explored the potential for the Internet of Medical Things (IoMT) and point-of-care (POC) devices to improve healthcare in advanced areas such as cardiac measurement, cancer diagnosis, and diabetes management. The review also assessed the significance of AI in improving the functionality, detection accuracy, and decision-making capabilities of IoMT devices while identifying potential risks. J. B. Awotunde et al. [181] suggested a framework based on AIoMT to monitor and diagnose patients and evaluate the model’s accuracy. Integrating AI and IoMT in the healthcare industry can alleviate the burden of medical processes and significantly enhance disease diagnosis, prediction, treatment, screening, and medication. The article by Y. Yang et al. [182] discusses potential research directions in medical research and proposes a smart health management valuable model for decision-makers and healthcare workers in hospitals.

Smart health monitoring systems (SHM) are effective for maintaining a healthy lifestyle amidst busy work schedules. With smart and cost-effective sensors developed through Industry 5.0 and 5G, health monitoring services have become faster, cost-effective, and reliable from remote locations [183]. In a systematic review, Z. F. Khan and S. R. Alotaibi [184] discussed the application of AI and big data analytics in improving the m-health system and proposed a model based on AI and big data analytics for m-health. Their model provides insights to users and enables them to plan and use resources effectively. S. Tian et al. [185] emphasized the potential of AI in drug discovery and its ability to accelerate the process, reduce costs, and improve accuracy. The researchers suggest that AI can transform drug discovery by identifying new drug candidates, predicting their efficacy and toxicity, and optimizing the drug development process. In their study, B. Zhou et al. [186] used NLP techniques to analyze various healthcare domains, such as hospital management, clinical practice, public health, personal care, and drug development. They emphasize the significance of NLP in facilitating smart healthcare and its potential to revolutionize how healthcare is delivered and improve patient outcomes. S. Saif et al. [187] explored the role of the IoT and AI in smart healthcare. They review recent developments in the field, including case studies on drug discovery and predicting chronic diseases such as heart disease and kidney-related ailments using ML and DL techniques.

#### 5.2.6. Smart Environment

The impact of AI on smart environment in a smart city scenario is significant. AI can help optimize energy usage, reduce waste, and enhance sustainability. For example, artificial intelligence algorithms can monitor and manage energy consumption in buildings, automatically adjusting lighting and temperature based on occupancy patterns, which can lead to significant reductions in energy waste and improved energy efficiency. AI can also help in waste management by predicting waste accumulation, identifying the optimal time for waste collection, and reducing transportation costs. AI can also be utilized for environmental monitoring and conservation. It can help track air and water quality, monitor vegetation changes, and predict natural disasters. Integrating AI and IoT devices makes it possible to create a more comprehensive and real-time picture of the environment, which can aid in environmental management and conservation efforts. AI can transform smart environment solutions in a smart city scenario. By leveraging AI, smart cities can become more sustainable, efficient, and resilient while improving citizens’ quality of life. Smart environment refers to using advanced technologies and data-driven solutions to improve natural and built environments’ sustainability, efficiency, and resilience.

G. Halhoul Merabet et al. [154] comprehensively examined methods utilizing AI to develop control systems. To reduce energy consumption while maintaining comfortable indoor conditions. L. Li et al. [155] discussed the obstacles encountered when applying AI to enhance energy efficiency and comfort, along with proposed paths for future advancements for research in this area. Their research describes developing and applying A versa-tile AI algorithm with multiple goals that can be implemented in real-world environmental control to quickly and precisely improve the Indoor Air Quality (IAQ), thermal comfort, and energy efficiency of buildings. The results showed a high level of accuracy in predicting outcomes, with over 90% accuracy. Additionally, there was a decrease in air pollution, an increase in thermal comfort, and an average energy savings of 31%, 45%, and 35%, respectively. A. I. Dounis [156] explored the possibility of using AI as a means of designing building automation systems and with an emphasis on the importance of optimizing energy usage, ensuring comfort, promoting good health, and enhancing productivity in residential areas. L. Anthopoulos and V. Kazantzi [157] discussed the increasing attention given to energy efficiency, especially in cities, in response to sustainability and climate change challenges. It highlights the impact of emerging technologies such as blockchain and electricity and suggests analyzing how autonomous vehicles, intelligent building systems, AI, and big data impact the energy system in urban areas. It also proposes a unified evaluation model for determining the energy efficiency of AI and big data. S. Rubab et al. [158] presented a comparative study of these techniques and suggested a comprehensive approach to managing COVID-19 waste, taking into account its potential uses and benefits and outlining both short-term and long-term goals for waste management. A. Shreyas Madhav et al. [159] offered the use of a mobile robot that can identify and segregate ordinary electronic wastes from households. The robot uses a convolutional neural network-based identification system to categorize E-wastes with 96% accuracy.

S. Shukla and S. Hait [160] examined how integrating technologies such as IoT, AI, intelligent transportation systems, and cloud computing could be utilized to establish intelligent waste management practices in smart cities within the current waste management systems. Y. Himeur et al. [161] discussed applying data fusion strategies and AI in evaluating and monitoring the environment, utilizing remote sensing (RS) data and satellite imagery. The discussion culminates in suggestions for future directions and recommendations. K. Bakker [162] discussed introducing a new technique for marine governance and environmental monitoring called AI-enabled, mobile marine protected areas (MMPAs). MMPAs use digital devices to gather information from different sources and employ ML algorithms to analyze the data and swiftly adjust to changes in environmental conditions and disruptions. S. J. Soheli et al. [163] discussed the capacity for enhancing crop production by utilizing an adaptive neuro-fuzzy inference system (ANFIS) and the IoT in a smart, automated greenhouse. The system used sensors to gather real-time data on weather factors such as temperature, humidity, sunlight, and soil moisture. Overall, the system is efficient, cost-effective, and has the potential to improve crop production in the greenhouse significantly.

D. Pamucar et al. [164] discussed the potential impact of Metaverse technologies on the transportation system, highlighted the preparations underway for the transition of transportation into the world of Metaverse, and concluded that Metaverse technologies could revolutionize the transportation system. The assessment framework can help decision-makers prioritize the implementation of sustainable transportation alternatives in the Metaverse. I. Ahmed et al. [165] introduced an intelligent and environmentally conscious theoretical model that uses cloud computing, IoT devices, and AI to handle and acquire essential data. The setup furnished digital data analysis and stores findings in decentralized cloud repositories using blockchain technology to support diverse applications. E. K. Nti et al. [166] discussed that the importance of AI could not be overstated when tackling environmental sustainability problems such as energy, water management, bio-diversity, and transportation. AI models, such as decision support and CV, have been used in transportation. However, it is vital to monitor interventions to enhance environmental sustainability continuously.

#### 5.2.7. Smart Living

Smart living refers to using advanced technologies and data-driven solutions to enhance living standards, ease of use, and the long-term viability of daily living. W. Li et al. [167] examined the development of smart home studies in the last twenty years, driven by technological advancements such as ICTs, AI, and IoT. IoT is prevalent in creating operational smart homes, yet there is an inadequate exploration of its impact on urban environments and societal concerns. Smart homes have the potential to become a significant contributor towards the realization of smart cities. X. Guo et al. [168] reviewed and aimed to comprehend the direction of smart home advancements and merchandise and the correlation between literature and AI-enabled products in smart homes. D. N. Mekuria et al. [169] discussed the difficulties of logical thinking in environments that supported assisted living technology and emphasized the significance of employing hybrid reasoning methods to manage multiple inhabitants’ overlapping, simultaneous, and conflicting pursuits and objectives. L. M. Gladence et al. [170] summarize that Smart Living Home automation is a technology that enables remote access and management of different devices and appliances within a household. IoT, AI, and cloud computing technologies can be utilized. This technology offers a more comfortable and convenient lifestyle for the physically impaired or elderly.

K. Zvarikova et al. [171] review literature from various databases, identify 18 main empirical sources, and show that AI chatbot customer service and hyper-realistic personalized interactive experiences can boost customer engagement. Additionally, the study suggests that digital interactive experiences involving virtual goods and assets influenced consumer behavior by utilizing location data. R. Dr. Bharati [172] highlighted the Chat Generative Pre-trained Transformer (ChatGPT), which facilitates the processing of natural language and generation of automated text, and its capacity to transform how humans interact and communicate with machines. W. Wang et al. [173] highlight the potential of using IoT, 5G, and AI technologies to revolutionize the tourism industry and provide personalized recommendations to travelers.

O. Taiwo et al. [174] presented the importance of security in smart homes and gave an intelligent home automation system that monitors environmental conditions, manages household appliances, and detects motion within the house and its surroundings. It highlights the significance of intelligent monitoring and response for the efficiency and safety of smart home automation. K. Ahmad et al. [175] explore the challenges of implementing data-driven AI services in modern smart cities, focusing on these algorithms’ security, safety, and interpretability and advising on existing constraints, drawbacks, and future avenues for research in these areas. M. H. Panahi Rizi and S. A. Hosseini Seno [176] recognize existing security and privacy remedies, unresolved research issues and obstacles, and furnish a structured literature map. The study aims to deliver a baseline for future research in the field and condenses the results into a collection of previously diverse and intricate evidence.

K. Jokinen et al. [177] presented the development of a virtual coach designed to assist older adults with everyday activities and help maintain their well-being and healthy lifestyles. The e-VITA EU-Japan Virtual Coach for Smart Aging project aimed to prototype virtual coaches for smart environments, develop standards for component interoperability, and conduct a study of “proof-of-concept” for assessing user acceptance. N. Bagaria et al. [178] reviewed existing literature on Health 4.0 Using Digital Twins for maintaining personal health and wellness, which involves essential terms, uses, and research gaps. The aim is to give a summary of the possible advantages and difficulties associated with the utilization of Digital Twins in the fields of healthcare and well-being. A. El Saddik et al. [179] also proposed a DTwins ecosystem that is going to accelerate the convergence of IoT, big data, communications mechanisms, security, and multimodal interaction to empower individuals’ wellness. It also enhances their quality of life. M. A. Makroum et al. [180] reviewed 89 studies published between 2011 and 2021 and concluded that wearable technology had gained significant attention in the medical field for individuals with long-term health conditions such as diabetes and has the potential to assist in diabetes management, prevent complications, and improve quality of life. Table 5 provides a summary of the major applications and the impact of AI on each of these applications.

## 6. Future Trends of Smart City

To manage different aspects of urban life, including but not limited to traffic, energy consumption, waste management, and public safety, a smart city requires extensive use of IoT devices for monitoring and control. These devices generate massive amounts of data that must be transmitted over the internet to the central control system for processing and analysis. Therefore, a gigantic IoT connection is essential for successfully implementing a smart city. Implementing a 5G-based [216] IoT network is a potential solution for managing a massive network in a smart city scenario. This network [217,218], based on the wireless software-defined networking (WSDN) paradigm, offers a flexible and rapid network infrastructure to facilitate the deployment of IoT-based smart city networks [219]. It has increased speed and bandwidth where the data transfer rate reaches up to 20 Gbps. The 5G network can manage the enormous volume of data IoT devices generate. Its higher bandwidth can accommodate more connected devices, enabling various new applications for enhancing urban life in smart cities.

**Figure 8:**
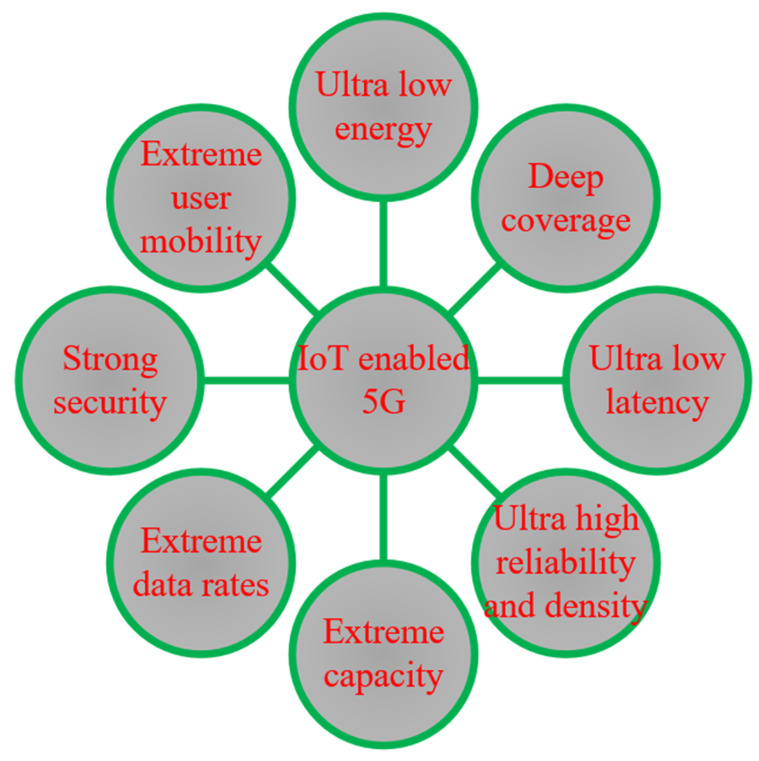
Probable characteristics of IoT-enabled 5G networks that would be useful for smart cities.

Additionally, 5G networks offer low latency and improved reliability, making them desirable for powering IoT-based smart city networks. With latency as low as 1 ms, 5G technology can enable the development of more real-time applications. The reduced latency ensures minimal delay between IoT devices, leading to faster and more efficient communication. This feature is crucial for developing applications such as smart drone transport, autonomous vehicles, real-time remote surgery, or patient monitoring for smart healthcare in a smart city. Devices with lower latency can send and receive data much more quickly, enabling a range of innovative and time-sensitive use cases. Compared to previous generations (i.e., 2G, 3G, or 4G), 5G networks are considered more reliable, offering features such as network slicing and multi-access edge computing (MEC). These features ensure consistent and uninterrupted connectivity for IoT devices in congested areas of a smart city. As the development of smart cities continues, 5G networks are expected to be increasingly used due to their ability to provide enhanced security features, such as secure boot, network slicing, and secure element, which can help prevent cyber-attacks and data breaches, ensuring the security of IoT devices and the massive data they generate (as depicted in Figure 8).

The impact of 5G on connecting billions of devices in a single communication technology is significant, especially in creating a massive IoT-enabled smart city. To achieve this, smart devices must be able to interact with each other and share data without the need for human intervention [220]. It is also vital for these smart devices to support real-time, demand-based events that can be coordinated between end-to-end devices. Additionally, they should be equipped with automatic and intelligent algorithms that can operate seamlessly during each phase of the smart city development, from planning to deployment and maintenance. This is going to ensure that the smart city infrastructure is efficient, scalable, and can adapt to the changing needs and demands of the citizens [221]. IoT-enabled 5G architectures are designed to provide a common platform that can fulfil the following requirements:

- 5G networks should be adaptable and independent based on the specific needs of the applications.
- Cloud-based Radio Access Network (CloudRAN) should be used to support massive connections of devices for various applications (as shown in Figure 6).
- The core network architecture should be easy to implement and provide on-demand functions for network configurations.

In the future, smart cities are going to rely heavily on 5G communications and advanced IoT technologies, which involve storing and uploading massive amounts of data to an IoT cloud server to extract useful information using data analysis methods. These smart cities are going to incorporate various aspects such as smart traffic, smart homes, smart agriculture, smart grids, and smart management. 5G-enabled IoT networks are going to provide multiple features, including direct device-to-device communication, typical architecture, advanced spectrum sharing, and interference management, which are going to be vital in building a complete smart city. Integrating AI with 5G networks is going to be integral to the future smart city. AI is going to handle massive amounts of data from IoT-based applications, such as public transportation, traffic lights, and citizens’ daily activities, for more accurate and precise decision-making. This is going to enable finding insights into data patterns that can be used to boost the efficiency and productivity of municipal government operations while reducing related expenses. Additionally, AI is going to help reduce human errors, make automated data-driven decisions, implement efficient urban management, and explore new commercial possibilities.

The role of AI in a smart city is to manage and analyze the vast amounts of data generated by IoT devices with accuracy and precision to aid decision-making. For example, public transportation, traffic lights, and citizens’ daily activities generate enormous amounts of data, and AI can identify insights that can increase the efficiency and productivity of municipal government operations while reducing costs. Furthermore, AI can minimize human errors, make automated data-driven decisions, implement efficient urban management, and explore new commercial possibilities. Using AI and 5G technology, energy providers can optimize renewable energy sources such as solar and wind power. AI can predict when the sun is going to shine or the wind is going to blow, and 5G can instantly communicate this information to energy providers, allowing them to adjust their energy supply accordingly. AI and 5G can also help reduce traffic congestion and emissions in the transportation sector by optimizing traffic flow and identifying the most efficient vehicle routes. This can decrease time spent on the road, which in turn can reduce emissions and save energy. Integrating AI and 5G technology can revolutionize how we manage energy and transportation, reducing carbon emissions and moving us towards a more sustainable future.

**Figure 9:**
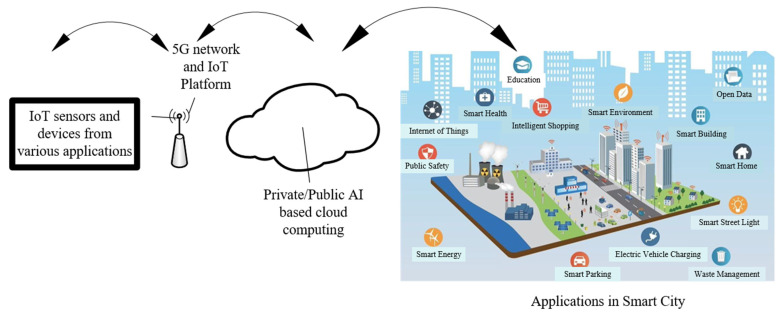
Illustration of the combination of IoT, 5G and AI, which would be the integral components of a futuristic smart city.

In conclusion, the success of a smart city relies heavily on its ability to integrate cutting-edge technologies such as 5G communication, IoT devices, and AI algorithms into its infrastructure. By working together, these technologies can create a more efficient, sustainable, and livable city. By integrating AI algorithms with renewable energy sources such as solar and wind power, cities can optimize energy usage and reduce carbon emissions, making the city more environmentally sustainable while reducing energy costs for residents and businesses. By leveraging these technologies, smart cities can become more efficient, productive, and ecologically sustainable, ultimately enhancing the quality of life for citizens. By reducing congestion, improving air quality, and optimizing the use of resources, smart cities can create a better living environment for all, making them an exciting prospect for the future of urban development. Figure 9 illustrates a futuristic smart city that combines IoT, 5G, and AI.

## 7. Conclusions

Communication technologies are essential to ensure reliable and continuous connectivity in smart cities. However, the proliferation of smart devices can present difficulties. Therefore, current research explores the potential of communication technologies enabled by the Internet of Things (IoT) and their possible applications in smart cities. Wi-Fi, ZigBee, and Z-Wave are suitable for short distances where devices and their coverage areas are limited, as they offer higher data throughput rates than long-distance technologies such as LoRaWAN. However, the choice of communication technology depends on the specific requirements of each application and the range of coverage needed. ZigBee is commonly used for low-power applications with a low data rate, providing a secure network and longer battery life. It offers network topologies suitable for various applications, such as mesh, star, and tree. On the other hand, some long-range technologies require devices to be connected through a central gateway to collect information and sink the data. These technologies include LoRaWAN, NB-IoT, Sigfox, and LTE, and they operate on licensed and unlicensed spectrums, depending on the applications. When choosing a communication technology, not only the low-power spectrum is essential, but scalability, network capacity, security, and regularity must also be considered.

Cellular networks can be a valuable resource for deploying new applications in a smart city. Narrowband IoT (NB-IoT) is a technology that focuses on low-cost devices while ensuring high network security and reliability. It also takes advantage of existing cellular networks, which can reduce the cost of installing a network for new smart city applications. On the other hand, LoRaWAN and Sigfox are unlicensed spectrum technologies that can help overcome the cost issues associated with licensed technologies. Depending on the application and region, these technologies typically use ISM bands, including 2.4 GHz, 868 MHz, 915 MHz, and 433 MHz. Sigfox is particularly useful for low-data applications such as smart environments, smart agriculture, and smart retailing. It claims to connect a million devices within a large coverage area.

Utilizing several AI algorithms in smart cities can enhance efficiency, sustainability, and quality of life. The algorithms discussed include ML, DL, NLP, CV, and RL. ML algorithms enable machines to identify patterns, learn from data, and make predictions based on input. On the other hand, DL models utilize artificial neural networks to understand hierarchical representations of data, allowing them to detect complex features and patterns. NLP algorithms enable computers to comprehend, interpret, and create human language, while CV algorithms enable machines to examine and understand visual data, recognize image patterns and features, and leverage this information to make informed decisions and predictions. RL is a machine learning algorithm in which an agent interacts with an environment through trial and error to maximize a cumulative reward. These algorithms can be used in various applications in smart cities, such as traffic management, energy optimization, waste management, and public safety. They can make smart cities more efficient, sustainable, and safer for residents.

The future of the smart city is going to depend on the development of every sector, such as a smart environment, smart agriculture, smart economy, smart business, and smart governance. The ability to incorporate the latest 5G communication technology, IoT devices, and AI algorithms into a city’s infrastructure is going to play a crucial role in the success of a smart city. Integrating these technologies can harmonize to produce a more efficient, sustainable, and livable city. Combining all the sectors can help build a smart city; this review explains the requirements. The available data transfer standard developed for IoT networks is not compatible. Therefore, lots of work must be conducted to enable intercommunication between the sensor nodes using different communication protocols while operating under low power. Another area the researchers or stakeholders must focus on is developing efficient storage techniques and low-power hardware design to reduce operational costs. Heterogenous networks for individual applications should be processed in one giant smart city network, and 5G can play a vital role in future smart city concepts. AI also has an enormous possibility for future work, including developing data fusion techniques for making heterogeneous data sources more accessible and intelligent data reduction for ensuring that surplus or ’uninteresting’ data are not part of the AI development pipeline. In summary, the current communication technologies are inadequate in providing uninterrupted connectivity in smart cities as they were initially created to accommodate a restricted number of devices and possess limited communication capabilities. Thus, there is a pressing need to develop intelligent and standardized protocols, such as the Internet of Things enabled by 5G technology, to cater to the needs of future smart cities effectively.

The current review paper has not thoroughly examined the ethical, social, and political ramifications of integrating AI with IoT in smart cities, including its potential impact on privacy, security, and social justice. Additionally, it lacks a comprehensive critical analysis of the advantages and drawbacks of this integration, leaving unanswered questions regarding the long-term sustainability, scalability, and efficacy of such approaches in smart cities.

## Figures and Tables

**Table 1:** Various definitions and concepts of the term ‘smart city’.

Definition of Smart City	Reference
The concept of a smart city involves leveraging ICT technologies to improve the performance of cities by enhancing the efficiency of municipal services, monitoring urban infrastructure, and supporting business development in both the public and private sectors.	[58]
A smart city is characterized by its innovative sociotechnical and socioeconomic development features, which aim to enhance the city’s intellectual capacity. The smart city concept also includes environmental protection and safety, often called a “green city”.	[59]
A smart city commonly employs Information and Communication Technology (ICT) to tackle the challenges of education, social welfare, and environmental sustainability.	[60]
A smart city is a community that incorporates advanced technologies, sustainable practices, and a safe and attractive environment, all interconnected to enhance its overall performance.	[61]
Integrating knowledge-intensive and creative approaches can enhance cities’ performance regarding socioeconomic development, environmental sustainability, and logistics.	[62]
Smart cities are designed to make communities healthier and happier under the situations that economic, social, and environmental models may offer.	[15]
A smart city employs various available technologies and resources intelligently to improve the sustainability and livability of urban areas.	[63]
A smart city is an urban area that leverages state-of-the-art technologies and creative approaches to boost sustainability, cleanliness, and the well-being of its inhabitants. Smart cities aim to enhance residents’ overall quality of life by integrating intelligent systems and data-driven solutions.	[64]
A smart city is a specific region that utilizes a holistic approach by integrating advanced technologies and real-time data analysis to facilitate sustainable economic growth.	[65]
Smart cities are highly productive, benefiting from educated people, knowledge-intensive careers, well-planned systems, and innovative events.	[66]
A smart city is a region with a strong capacity for learning and innovation, leveraging the creativity of its residents, knowledge institutions, and intelligent infrastructure for efficient communication and management.	[67]
Smart cities leverage information and communication technologies to optimize their infrastructure, resulting in increased convenience, efficiency, energy conservation, and environmental sustainability. Additionally, these cities have advanced monitoring systems that enable real-time detection and swift resolution of issues, efficient disaster recovery, and comprehensive data collection for informed decision-making.	[4]
Communication and sensor technologies offer smart cities significant advantages in enhancing logistical operations and ultimately enhancing the well-being of their residents.	[68]
A city can be considered smart when it effectively leverages its human and social resources and a combination of traditional and innovative communication infrastructure to support sustainable economic growth and enhance its residents’ overall quality of life while responsibly managing its natural resources.	[54]

**Table 2:** Evolution of Cellular communication technologies for M2M [94,95].

	Spectrum (MHz)	Channel Width	TX Rate DL	TX Rate UL	TX Power UL	Duplexing	Availability
Release 8 Cat-4	700–900	20 MHz	150 Mbps	50 Mbps	23	Full Duplex	Available
Release 8 Cat-1	700–900	20 MHz	10 Mbps	5 Mbps	23	Full Duplex	Available
Release 12 Cat-0	700–900	20 MHz	1 Mbps	1 Mbps	23	Half Duplex	Available
Release 12/13 LTE-M	700–900	1.4 MHz	200 kbps	200 kbps	20	Half Duplex	2016
Release 13 NB LTE-M	700–900	200 kHz	200 kbps	144 kbps	23	Half Duplex	2016
EC-GSM	800–900	200 kHz	~300 kbps	≤10 kbps	23–33	Half Duplex	2016
CS IoT	700–900	5kHz (UL) 3.75 kHz (DL)	200 kb/s	~48 kbps	≤23	Half Duplex	2016

**Table 3:** A comparison of different short-range communication standards.

		Spectrum	Channel Width	Tx Rate Uplink	Tx Rate Downlink	Packet Size	Max Range (km)	Tx Power	Standard (If Any)
Sigfox		868–902 MHz	100 Hz	≤100 bps	256 bpday	≤12 bytes	10–50	10 μW–100 mW	Available
LoRaWAN	EU	863–870 MHz & 433 MHz	125–250 kHz	250–50 bps	250 bps–50 kbps	≤222 bytes	2–15	14 dBm	Available
	US	902–928 MHz	125–500 kHz	980 bps–21.9 kbps	980 bps–21.9 kbps	≤222 bytes		20 dBm	Available
Weightless	W	470–790 MHz TV white spaces	6–8 MHz	250 bps–50 kbps	2.5 kbps–16 Mbps	≥10 bytes	5	17 dBm	Available
	N	Sub GHz (ISM)	200 Hz	250 bps	None	≤20 bytes	3	17 dBm	Available
	P	Sub GHz (ISM)	12.5 kHz	200 bps–100 kbps	200 bytes–100 kbps	≥10 bytes	2	17 dBm	Available
Ingenu		2450 MHz	1 MHz	624 kbps	156 kbps	6 bytes–10 k bytes	100	20 dBm	Available

**Table 4:** Short-range communication standards for multi-tier IoT architectures.

	ZigBee (802.15.4)	WI-SUN (802.15.4g)	ULP (802.15.4q)	Wireless M-Bus	Z-Wave	Bluetooth Low Energy (BLE)	Wi-Fi Low Power (802.11ah)
Frequency bands	868/915/2450 MHz	Sub-1 GHz, 2.4 GHz	868/915/2450 MHz	169/433/868 MHz	908 MHz	2.4 GHz	Sub-1 GHz
Max Tx rate	250 kbps	1 Mpbs	100 kbps	100 kbps	100 kbps	1 Mbps	7.8 Mbps
TX power	1–100 mW	1–100 mW	5–15 mW	1–100 mW	1–100 mW	1–100 mW	10 mW–1 W
Max range (m)	100	200	100	300	100	30	1000

**Table 5:** Summary of the impact of AI on smart city technologies.

Major Domains	Impact of AI	Reference
Smart Mobility	Improved traffic management	[143,144,145,146]
	Autonomous vehicles	[147,148,149,150]
	Predictive maintenance	[151,152]
Smart Governance	Improving decision-making	[153,154,155]
	Enhancing public services	[156,157]
	Increasing transparency and accountability	[158,159,160,161]
Smart Education	Personalized learning	[162,163,164]
	Intelligent tutoring	[165,166,167]
	Curriculum development:	[168,169,170]
Smart Economy	Increased productivity	[171,172,173,174]
	Improved decision-making	[175,176]
	Enhanced customer experience	[177,178,179]
Smart Healthcare	Medical diagnosis	[180,181,182]
	Predictive analytics	[183,184,185,186,187,188]
	Drug discovery	[185,186,187]
Smart Environment	Energy efficiency	[189,190,191,192]
	Waste management	[193,194,195]
	Environmental monitoring	[196,197,198]
	Sustainable transportation	[199,200,201]
Smart Living	Home automation	[202,203,204,205]
	Personalized recommendations	[206,207,208]
	Security and safety	[209,210,211]
	Health and wellness	[212,213,214,215]

## Data Availability

Not required.
